# The Psychiatric Patient as a Health Resource Consumer: Costs Associated with Electroconvulsive Therapy

**DOI:** 10.3389/fpsyg.2016.00790

**Published:** 2016-05-27

**Authors:** Carmen Selva-Sevilla, Maria Luisa Gonzalez-Moral, Maria Teresa Tolosa-Perez

**Affiliations:** ^1^Department of Applied Economics, University of Castilla-La ManchaAlbacete, Spain; ^2^Department of Clinical Analysis, University Hospital Complex of AlbaceteAlbacete, Spain; ^3^Department of Psychiatry, University Hospital Complex of AlbaceteAlbacete, Spain

**Keywords:** mental disorders, electroconvulsive therapy, patient satisfaction, costs and cost analysis, costs of health care, direct service costs

## Abstract

**Background:** Clinical practice protocols should consider both the psychological criteria related to a patient’s satisfaction as a consumer of health services and the economic criteria to allocate resources efficiently. An electroconvulsive therapy (ECT) program was implemented in our hospital to treat psychiatric patients. The main objective of this study was to determine the cost associated with the ECT sessions implemented in our hospital between 2008 and 2014. A secondary objective was to calculate the cost of sessions that were considered ineffective, defined as those sessions in which electrical convulsion did not reach the preset threshold duration, in order to identify possible ways of saving money and improving satisfaction among psychiatric patients receiving ECT.

**Methods:** A descriptive analysis of the direct health costs related to ECT from the perspective of the public health system between 2008 and 2014 was performed using a retrospective chart review. All of the costs are in euros (2011) and were discounted at a rate of 3%. Based on the base case, a sensitivity analysis of the changes of those variables showing the greatest uncertainty was performed.

**Results:** Seventy-six patients received 853 sessions of ECT. The cumulative cost of these sessions was €1409528.63, and 92.9% of this cost corresponded to the hospital stay. A total of €420732.57 (29.8%) was inefficiently spent on 269 ineffective sessions. A sensitivity analysis of the economic data showed stable results to changes in the variables of uncertainty.

**Conclusion:** The efficiency of ECT in the context outlined here could be increased by discerning a way to shorten the associated hospital stay and by reducing the number of ineffective sessions performed.

## Introduction

An economic evaluation of health care is needed because financial resources are limited and must therefore be allocated in the most efficient way. In the field of health, such evaluations would prove useful because they would help decision-makers identify the best ways to allocate resources, which would be immensely helpful for making decisions both at the general level of health by Health Administration and at a more specific level by health staff (Drummond et al., 2015).

In recent years, there has been an ongoing discussion regarding the concept of “patient-centered care”; this concept considers allowing patients to play an important role in making decisions about their own health. Furthermore, clinical practice decisions should consider not only factors related to the effectiveness and efficiency of a treatment but also the perspective of the patient, specifically his/her satisfaction with the treatment process (Mira and Aranaz, 2000).

The subject of this work is the more specific case of the psychiatric patient, for whom satisfaction with the care process is evaluated by questionnaires fulfilled by both the patient and his/her relatives. These questionnaires have identified the most important items that influence a positive perception of health service by the patient. Those items include (1) the competence of care professionals, (2) being given complete information about the diagnosis and treatment, (3) patient involvement in the treatment plan, and (4) providing an explanation for the usefulness of the hospitalization (Berghofer et al., 2001; Blenkiron and Hammill, 2003; Gani et al., 2011; Fernandez-Carbonell et al., 2012).

Electroconvulsive therapy (ECT) is currently considered an effective treatment for patients with severe psychiatric disorders, especially for severe depressive disorders (mainly psychotic depression), some cases of acute mania and schizophrenic disorders (Bernardo, 1999; American Psychiatric Association Committee on Electroconvulsive Therapy, 2001; Rodriguez-Jimenez, 2015a). ECT consists of the application of electrical stimulation to the brain in order to trigger a generalized seizure. It is accepted that the optimal response to ECT is related to the duration of the triggered seizures, although there is no consensus for what the limit should be. ECT is administered two or three times per week for the acute phase of the disease, until between 6 and 12 sessions have been administered; usually, acute phase treatment is performed on an in-patient basis (Sackeim, 1991; Bernardo, 1999; American Psychiatric Association Committee on Electroconvulsive Therapy, 2001; Ding and White, 2002; Gonzalez et al., 2007; Perestelo-Perez et al., 2013; Rodriguez-Jimenez, 2015a). After completing the acute phase, the patients may possibly continue to receive additional ECT sessions as part of a continuation cycle or a maintenance cycle, depending on whether these sessions are applied during the first 6 months after the acute phase (continuation cycle to prevent relapses) or after 6 months (maintenance cycle to prevent recurrence; American Psychiatric Association Committee on Electroconvulsive Therapy, 2001). However, it is a more common practice to indiscriminately call them continuation/maintenance (Rodriguez-Jimenez, 2015a).

An ECT program was implemented in our hospital to expand treatment alternatives beyond drug treatment. Regarding the Spanish health care system, economic evaluations focused on ECT are scarce, and to the best of our knowledge, there has been no evaluation of the economic impact of the so-called ineffective sessions. The aim of this work was to identify possible ways of optimizing the use of health and economic resources, which in turn would improve patient perception of the treatment process.

Specifically, the main objective of this study was to determine the cost associated with the sessions of ECT that were implemented in our hospital between 2008 and 2014. A secondary objective was to calculate the cost of sessions that were considered ineffective, defined as those sessions in which the electrical convulsion did not reach the preset threshold duration.

## Materials and Methods

A retrospective chart review of all patients who had received at least one ECT session from April 2008 to December 31st, 2014 while admitted at the Mental Health Service in a 752-beds tertiary hospital was conducted. No exclusion criteria were considered.

A partial economic evaluation consisting of a description of costs was done from the perspective of the public health system. Only direct health costs were considered for our analysis, which include those costs incurred by the health system as a direct result of the disease (Oliva et al., 2015).

The cost of a standard ECT session was calculated as the product of health resources employed (measured in natural units and obtained from the routine records of each ECT session) for their unitary prices (measured in monetary units of the 2011 base year, discounted at a rate of 3%). The cost of an ECT session for each patient and each given cycle (the acute phase versus the continuation/maintenance phase) was also calculated. For this purpose, the cost of the hospital stay associated with ECT, the cost of the time spent by the medical staff, the consumables and drugs employed, and the cost of the purchase of the ECT equipment were considered, with the following observations:

### Hospital Stay

Information about the costs of a 24-h hospital stay at the Department of Mental Health was expressed in euros per day and was provided by the Control Service Management of our hospital. The total hospital stay was obtained from patients’ charts. For the purpose of this study, three different time periods of hospitalization were defined: pre-ECT, ECT, and post-ECT. The total days of admittance to the Mental Health Department were calculated by subtracting the date of hospital discharge from the date of hospital admittance. The days attributable to the ECT period were calculated by subtracting the date of the last ECT from the date of the first ECT session and then adding 2 days to account for anesthetic evaluation. If the patient had been transiently discharged during the weekend holidays, then these days were subtracted from the days attributable to the ECT period.

### Medical Staff and Nursing

Information about the salary (expressed in euros per minute of time spent by the professionals involved) was provided by our Service Management Control. To calculate the cost per staff caused by this activity, a typical ECT session was considered to be 30 min of time consuming activity for the following professionals: three physicians (two from the Mental Health Department and one from the Anesthesiology Department) and three nurses (two from the Anesthesiology Department and one from the Mental Health Department). The time spent by other professionals, such as assistants and orderlies, was not considered for this analysis.

### Drugs and Consumables

Information concerning the acquisition cost of drugs and consumables used in an ECT session according to our protocol was provided by the Hospital Pharmacy and Management Control Services.

### Equipment

The equipment with which the ECT was applied was ABBOT SPECTRUM brand (model 5000Q, EQ-ELMED class), and it was acquired in December, 2007 for €23500. This information was provided by the Management Control Service. The equivalent annual cost (at an interest rate of 3% and considering a useful life of 10 years) was calculated since its acquisition, and this cost was divided by the number of annual sessions of ECT conducted in the study period.

The cost of all of the ECT sessions was calculated by adding the cost of each session received by all of the patients. The cost of the ineffective sessions was calculated in the same way, i.e., by adding the cost of each ineffective session of all patients. Following the established protocol of the Mental Health Department, ECT sessions were considered ineffective when an electrical convulsion lasted less than 20 s. A more conservative approach was taken to assess the cost of ineffective sessions, considering those sessions lasting 20 or more seconds after re-stimulation as not ineffective, and by also not considering titration sessions because titration sessions are used to determine the minimum threshold electrical charge for each patient in each acute phase cycle and are, therefore, associated with a higher percentage of ineffective sessions.

In addition, these cost calculations were repeated considering 25 s, not 20 s, as the minimal duration for an acceptable electrical convulsion because 25 s is the most frequently cited limit in the psychiatric literature.

A sensitivity analysis was conducted, considering variations from the base case, in terms of personnel costs, the discount rate and the average cost per hospital stay.

### Statistical Plan

This is a descriptive study in which continuous variables were described using means and standard deviations, and the qualitative variables were described using absolute and relative frequencies.

### Ethics Statement

This chart review study was approved by the Ethics Committee and Clinical Research of our hospital.

## Results

During the implementation of the ECT program at our hospital from April 2008 to December 2014, 76 patients received at least one ECT session, accounting for 853 sessions. **Table 1** shows the characteristics of the patients and the ECT sessions. For 13 sessions (1.5%), the information about seizure duration was not available, and the sessions were classified as effective to avoid bias favoring the economic burden of ineffective sessions (our second objective).

**Table 1:** Clinical and demographic characteristics of patients undergoing electroconvulsive therapy.

	*N*	%
Patients	76	–
Age (years)	52 ± 16	–
Sex (female)	46	60.5
Diagnostic indication for ECT		
Affective disorder without psychotic symptoms	27	35.5
Affective disorder with psychotic symptoms	17	22.4
Psychotic disorder	30	39.5
Others	2	2.6
Clinical outcome		
GCI-I = 1 (very much improved)	22	21.8
GCI-I = 2 (much improved)	40	39.6
GCI-I = 3 (minimally improved)	28	27.7
GCI-I = 4 (no change)	11	10.9
ECT sessions	853	–
Sessions with convulsion lasting <20”	269/853	31.5
Sessions with convulsion lasting <20” re-stimulated	164/269	61.0
Re-stimulated sessions with convulsion lasting ≥20”	73/164	44.5
Sessions with convulsion lasting <25”	426/853	49.9


*ECT, electroconvulsive therapy; GCI-I, Global Clinic Impression-Improvement scale.*

The cost of a standard ECT session was €1667.35. The distribution of the costs derived from a standard ECT session is shown in **Table 2**, where it can be seen that the hospital stay represented the main economic burden.

**Table 2:** Average cost of a standard electroconvulsive therapy session (2008–2014)^a.^

	Unitary costs	Health resources	Average cost (%)
HOSPITAL STAY^b^	665.11	2.34	1529.76 (91.8)
STAFF^c^			74.70 (4.5)
Physician salary	0.50	90	45.26 (2.7)
Nurse salary	0.33	90	29.44 (1.8)
EXPENDABLES			35.33 (2.1)
Nasal O_2_-cannula and CO_2_-sensor with female luer adapter connection^d^	19.11	1	19.11 (1.1)
Sensor for level of sedation and consciousness in anesthetic processes	15.94	1	15.94 (1.0)
3-piece syringe, concentric catheter cone 50 ml	0.17	1	0.17 (<0.1)
2-piece syringe, eccentric luer cone 20 ml	0.05	1	0.05 (<0.1)
2-piece syringe, eccentric luer cone 10 ml	0.03	1	0.03 (<0.1)
2-piece syringe, eccentric luer cone 5 ml	0.02	2	0.04 (<0.1)
EQUIPMENT^e^	20.61	1	20.61 (1.2)
DRUGS			6.95 (0.4)
Propofol 1% 200 mg	2.01	1	2.01 (0.1)
Remifentanil 1 mg	2.98	1	2.98 (<0.2)
Atropine 1 mg	0.10	1	0.10 (<0.1)
Succinylcholine 100 mg	0.31	1	0.31 (<0.1)
Dexketoprofen 50 mg	0.57	1	0.57 (<0.1)
Saline solution 0,9% 100 ml	0.44	1	0.44 (<0.1)
Sodium chloride solution 0.9% for irrigation, sterile 500 Ml	0.56	1	0.56 (<0.1)
TOTAL			1667.35 (100)


*^a^All costs are in euros of 2011 and are discounted at a rate of 3%. ^b^Cost per hospital stay is expressed in euro/day. ^c^Staff costs are expressed in euros/minute. ^d^These calculations correspond to the first session of each cycle because the nasal cannula is re-used in every electroconvulsive therapy session for the same patient. ^e^Cost of equipment has been obtained by calculating the Annual Equivalent Cost (at an interest rate of 3% and useful life of 10 years) and dividing it by the number of annual sessions of electroconvulsive therapy.*

The cumulative cost of all of the 853 ECT sessions administered was €1409528.63 (**Table 3**). Of those, €420732.57 (29.8%) was inefficiently employed for performing 269 ineffective sessions, defined as when the electrical seizure did not reach 20 s. Following our conservative approach, 31 titration sessions lasting less than 20 s with an associated cost of €52165.90 were excluded from the ineffective group, and there were also 62 sessions lasting more than 20 s after re-stimulation with an associated cost of €98671.55. Therefore, using a more conservative approach €269865.42 (19.1%) were determined to be used inefficiently.

**Table 3:** Total cost of electroconvulsive therapy sessions (2008–2014).

Total cost of ECT sessions	1409528.63
Hospital stay	1308884.56
Staff	54643.19
Expendables	25841.87
Equipment	15076.25
Drugs	5082.89
Total cost for sessions with electric convulsion lasting <20”	420732.57
Total cost for sessions with electric convulsion lasting <25”	681068.03


*ECT, electroconvulsive therapy. All costs are expressed in constant euros of 2011 and discounted at a rate of 3%.*

By pure logic, setting 25 s as the lower limit for an adequate electrical convulsion would increase the number of inefficient sessions (426, **Table 1**) and the cost associated to inefficient sessions (48.3%, **Table 3**).

As stated above, the hospital stay represented the largest portion of the total cost (92.9%; **Table 3**). Before it had been decided to treat the patients with ECT (pre-ECT period), they had been admitted for a median of 15.5 days (interquartile range 5–25.25), representing a cost of €891564.38. Considering this time course, the cost of treating psychiatric patients would have increased by more than 60%, specifically from €1409528.63 to €2301093.01. In contrast, during the ECT period, patients had 149 days of weekend discharges, resulting in a savings of €89102.57 (5.9%).

The sensitivity analysis of the economic data shows stable results for variations of the uncertainty variables (**Table 4**).

**Table 4:** Sensitivity analysis.

Base case	1409528.63
Staff, 20 min	1388629.26
Staff, 40 min	1430429.30
Discount rate 0%	1549554.62
Discount rate 5%	1348736.29
10% increase in hospital stay cost	1540417.06
10% decrease in hospital stay cost	1278640.15
20% increase in hospital stay cost	1671305.52
20% decrease in hospital stay cost	1147751.70


*Total cost of electroconvulsive therapy sessions (2008–2014). All costs are expressed in constant euros of 2011.*

## Discussion

The main results of our work are, first, that the hospital stay is responsible for more than 90% of the total costs attributed to ECT and, second, that there are a large number of ineffective ECT sessions, which implies that almost 30% of the economic resources had been inefficiently used.

Regarding the economic evaluation of ECT, there have been studies that have reported a cost-effectiveness analysis or cost description analysis in which ECT is compared with pharmacotherapy (Aziz et al., 2005; Greenhalgh et al., 2005) or other techniques such as repetitive transcranial magnetic stimulation (Kozel et al., 2004; McLoughlin et al., 2007; Knapp et al., 2008; Vallejo-Torres et al., 2014), as well as studies focusing on continuation/maintenance programs (Bonds et al., 1998; McDonald et al., 1998; Aziz et al., 2005; Odeberg et al., 2008; Rodriguez-Jimenez et al., 2015b). In general, these studies have concluded that ECT is cost-effective.

Based on this premise, we have conducted a purely descriptive study of the direct costs that our hospital has incurred after implementing an ECT program. According to our results, the cost of a standard ECT session was €1667.35. In our environment, Vallejo-Torres et al. (2014) found a cost of €737, but they only considered the costs of staff, materials, equipment, tests, and drugs in their analysis, and they did not include the cost of the hospital stay. If they had included this cost, then the cost of a standard ECT session would rise to €1475 (referred to in 2013 euros and ascribing 2 days of stay for each ECT session), a figure very similar to what we found.

In view of our results, we propose two ways to improve the efficiency of the ECT: reducing the number of ineffective sessions and shortening the hospital stay of the patients. By attaining these objectives, two advantages would be expected: first, an improvement in the efficiency of the treatment itself and, second, a consideration of the patients’ preferences in line with the above mentioned “patient-centered care.” By implementing these actions, patients would be allowed to return to their homes and resume their activities earlier, which, in turn, would be perceived with great satisfaction both for the patients and their relatives.

Regarding the objective of reducing the number of ineffective sessions, we found that the percentage of funds invested in ineffective sessions (those lasting less than 20 s; Bertolin-Guillen et al., 2006; Ramirez-Segura and Ruiz-Chow, 2013) ranged between 29.8 and 19.1%, as assessed either in a less or more conservative approach, respectively. A clear limitation of this assessment is the assumption that the clinical efficacy of ECT depends solely on the electrical length of the evoked seizure, when there is no evidence that this assumption is true (Fear et al., 1994; Geretsegger et al., 1998; Sackeim, 1999; Azuma et al., 2007; Bauer et al., 2009; Stewart, 2012; Martinez-Amoros et al., 2014). In fact, different values for the lower limit of the electrical convulsion can be found in the literature: 15 s (Gonzalez et al., 2007), 20 s (Bertolin-Guillen et al., 2006; Ramirez-Segura and Ruiz-Chow, 2013), 25 s (Nguyen et al., 1997; Dogan et al., 2011; Gombar et al., 2011; Hizli Sayar et al., 2014; Martinez-Amoros et al., 2014), and 26 s (Wang et al., 2011). These data preclude us from directly comparing the percentage of sessions that were considered ineffective. To perform an indirect comparison with other authors who used propofol as a hypnotic and to consider the lower limit of efficacy at 25 s, we re-analyzed our data after establishing the 25 s limit. Using this approach, our percentage of ineffective sessions would rise to 50%, a value that is much lower than that found by Martinez-Amoros et al. (2014; 82.9%) but much higher than that found by Nguyen et al. (1997; 4.2%). This huge disparity in the percentage of ineffective sessions could be explained by the small number of sessions held by these authors: 35 sessions (Martinez-Amoros et al., 2014) and 22 sessions (Nguyen et al., 1997).

However, despite the considerable number of so-called ineffective sessions that have taken place, the efficacy of ECT in our patients measured by the CGI-I was 61.4%. This finding was obtained as a result of the sum of cycles of treatment whose clinical development had been “very much improved” and “much improved,” as shown in **Table 1**.

Our percentage of ineffective sessions (19.1%, conservative estimate) suggests that there is room for improvement in the performance of the ECT sessions. This improvement would include modification of the guidelines of psychiatry and anesthesiology, which is beyond the scope of this work.

Regarding the second aspect of our investigation, we did a thorough analysis of the costs associated with ECT and found that micro-costs (i.e., those costs associated with consumables, equipment and drugs) could be ignored in future studies because they represent a practically negligible percentage of the total cost (less than 4%). Conversely, the hospital stay represented the highest percentage of economic burden; therefore, any strategy to save costs should be aimed at reducing the hospital stay of the patients.

We found an average delay of 2 weeks from hospital admittance to inclusion in the ECT program. It was not the aim of this work to study the costs associated to this pre-ECT period, but they were definitely significant costs assumed by the Health System and a clear target for some cost-saving measures. This delay occurs because it is routine clinical practice of our Mental Health Department physicians to exhaust drug therapy before entering the patients into an ECT program.

To reduce the hospital stay, one possibility could be to exhaust drug therapy in an out-patient setting for those patients whose clinical condition would permit out-patient management; therefore, in the event that they must be admitted to receive ECT, it could be performed soon after admittance. For those patients whose clinical condition would not allow for out-patient management before drug alternatives had been exhausted, ECT could be considered earlier than when it is currently indicated. In either case, the length of the hospital stay would be shortened (Kramer, 1990; Chan et al., 2006).

Another way to reduce the hospital stay would be by increasing weekend discharges. Until now, this option has been scarcely adopted. We did not review the full clinical aspects of the patients; thus, based on our data, it is impossible to determine whether more weekend discharges were possible for clinical reasons, which represents a clear limitation to our statement. However, weekend discharges in our Mental Health Department are not a standard protocol; therefore, we do believe that there is a potential to save costs in this way.

Another way to reduce the duration of hospital stays would be to assess the performance of ambulatory ECT (Kramer, 1990; Chan et al., 2006; Rodriguez-Jimenez, 2015a) instead of the traditional custom of performing ECT in an in-patient manner for the entire duration of the corresponding cycle. Selected patients, such as those not requiring ongoing psychiatric care, could benefit the obvious advantages of ambulatory treatment, such as staying at home with their relatives for as long as possible, which, in turn, could significantly contribute to improving their clinical condition and perhaps would also improve the patients’ perception of and satisfaction with their psychiatric treatment. Ambulatory ECT is already a real option in our context (Lopez Villaescusa et al., 2011; Rodriguez-Jimenez, 2015a).

This work has several limitations. Some of the limitations have been exposed above, for example, the assumption that the clinical efficacy of ECT depends solely on the electrical length of the seizure, not having analyzed the causes behind the delay between the hospital admission and the decision to indicate ECT, and not explaining the limited number of weekend hospital discharges of the patients. In addition to these limitations, this work has the general limitations of any retrospective review (i.e., a lack of information in medical records) and a more specific economic limitation, that is, that the average costs of the hospital stay (cost *per diem*) were used to assess the cost of the stay (Drummond et al., 2015).

## Conclusion

Electroconvulsive therapy efficiency could increase by reducing the number of ineffective sessions and by reducing hospital stay. Whether a reduction in the number of ineffective sessions could be attained by modifying the anesthesiologist-psychiatric protocol deserves future research, as well as a reduction in hospital stay by encouraging hospital permissions and ambulatory ECT. On the basis of these assumptions were true, the patients’ perceptions of their treatment process would be improved for three main reasons: clinically, because their treatment efficacy would be increased; psychologically, because their hospital admittance would be shortened in time or even avoided; and economically, because by applying the concept of opportunity cost, the economic savings could be employed to benefit the patients even if they are unaware of these savings.

## Author Contributions

CS-S has done the economical evaluation and has contributed to statistical analysis. MG-M has done the main part of statistical analysis. MT-P has reviewed the clinical charts. All authors have made substantial, direct and intellectual contribution to the work, and approved it for publication.

## Conflict of Interest Statement

The authors declare that the research was conducted in the absence of any commercial or financial relationships that could be construed as a potential conflict of interest.

The reviewer FSR and handling Editor declared their shared affiliation, and the handling Editor states that the process nevertheless met the standards of a fair and objective review.
